# Low prevalence of multi-resistant bacteria in undergraduate dental students; an observational case-control multi-centre study in Europe

**DOI:** 10.1080/20002297.2021.1889898

**Published:** 2021-02-21

**Authors:** C.M.C. Volgenant, M.A. Hoogenkamp, G. Dahlén, S. Kalfas, S. Petti, J.J. De Soet

**Affiliations:** a Department of Preventive Dentistry, Academic Centre for Dentistry Amsterdam (ACTA), University of Amsterdam and Vrije Universiteit Amsterdam, Amsterdam, The Netherlands; b Department of Public Health and Infectious Diseases, Institute of Odontology, Sahlgrenska Academy, University of Gothenburg, Sweden; c Division of Preventive Dentistry Periodontology and Implant Biology, Aristotle University of Thessaloniki, Thessaloniki Greece; d Department of Public Health and Infectious Diseases, Sapienza University, Rome, Italy

**Keywords:** (MeSH-terms): MDR/multi-drug resistant bacteria, dentistry, students, public Health, methicillin-Resistant Staphylococcus aureus (MRSA), extended Spectrum Beta-Lactamase (ESBL), vancomycin Resistant Enterococci (VRE)

## Abstract

**Objective:** This study assessed the prevalence of MRSA, ESBL and VRE in students from four dental schools in Europe.

**Methods:** The hand, tongue and nostrils of the students who treated patients (study group) and who did not treat patients (control group) were sampled. After incubation in TSB and subculturing in the presence of 4 µg/ml oxacillin, positive cultures were identified for *Staphylococcus aureus* by Mannitol salt agar and agglutination tests. The presence of MRSA was confirmed by specific PCR on the species and on the *SSCmec* genes. ESBL and VRE were isolated using specific CHROMagar and confirmed using antibiotic sensitivity tests.

**Results:** Of the 879 students who participated in this study (454 students which treated patients, 425 controls) a total of 50 students (5.7%) tested positive for a multi-drug resistant bacterium (MDRB); 13 (1.5%) students tested positive for MRSA, 26 (3.0%) for ESBL and 12 (1.4%) for VRE. No statistically significant differences were found between the students who treated patients compared to the control group for any of the MDRB and study centres, excluding MRSA carriage in the Italian student population. The use of antibiotics the year before sampling, was positively associated with the presence of an MDRB (OR 2.0; 95% Confidence Interval 1.10–3.68; p = 0.02).

**Conclusion**: The risk for MDRB carriage and sequential transmission of MDRB for dental health care students and their patients were acceptably low.

## Introduction

Transmission of pathogens is to be expected during a dental treatment [1]. Transmission of microorganisms from the oral cavity or the skin can take place from a patient to the dental team or vice versa, via direct contact, indirect contact or via aerosols. Little is known, however, about the clinical consequences of transmission of microorganisms during dental treatment. The recent coronavirus pandemic questions the extent of transmission of microorganisms to dental healthcare professionals (DHCPs) during dental treatment. SARS-CoV-2 can, apart from its transmission via aerosols and droplets [2], be transmitted via surfaces [3], which is similar to the transmission route for multi-drug resistant bacteria (MDRB).

An important theme within infection control is the transmission of MDRB with a large health burden [4]. Therefore, as in healthcare, the aim in the dental practice is to prevent the transfer of MDRB between patients and DHCPs, or vice versa. Most common MDRB are Methicillin-Resistant *Staphylococcus aureus* (MRSA), Extended Spectrum Beta-lactamase producing *Enterobacteriaceae* (ESBL) and Vancomycin Resistant Enterococci (VRE) [5]. The occurrence of these MDRB is a growing concern as, in case of an infection, these microorganisms are difficult to eradicate using standard antibiotics [6].

*S. aureus* is a commensal bacterium which resides in the nose, throat and oral cavity. MRSA has been isolated from a variety of oral infections [7]. In some countries DHCPs, which are tested positive for MRSA, are regarded as a potential risk of bacterial transmission to patients and colleagues. These DHCPs are, as a consequence, not allowed to treat patients as long as they are colonized with this bacterium [8 9, 10].

Previous research reported that the prevalence of MDRB carriers, within dental workers, is not higher than the prevalence in a normal adult population [11]. This is in contrast with another study which concluded that 21% of dental students carried MRSA in their nose, a prevalence ten times higher than the normal population and two times higher when compared to other university students [12]. Information on the occurrence of multi-resistant bacteria in European DHCP is not available. Moreover, no data are available on the transmission of VRE and ESBL-bacteria within the dental surgery.

This cross-sectional study is conducted to investigate whether dental students, who perform clinical procedures, are at risk of infection from MRSA, ESBL-bacteria and VRE. It is hypothesized that transmission occurs during patient treatment, especially in countries where the carriage prevalence of MDRB in the population is relatively high [13]. Therefore, this study was performed at universities from four European countries with different carriage rates of these MDRB [13]. The primary outcome of the study was to assess MDRB prevalence in clinical dental students compared to a control group. The secondary outcome was to assess which factors are associated with MDRB carriage, such as previous antibiotic use, living in rural areas, living near cattle, treatment of patients, hand hygiene protocols and the last application of hand hygiene.

## Methods

### Study design

A multi-centre cross-sectional study was conducted at four dental institutes in Europe: Sahlgrenska Academy (Gothenburg, Sweden), Aristotle University of Thessaloniki (Greece), Sapienza University (Rome, Italy) and the Academic Centre for Dentistry Amsterdam (ACTA, The Netherlands). From each dental school a group of dental students was invited to participate., Patient treatment status was recorded to determine whether the students performed patient treatment (study group) or only pre-clinical work (control group).

As students at the ACTA already have clinical patient contact in the first year, students of the 2^nd^ year of the Faculty of Life Sciences (Vrije Universiteit Amsterdam) and of the Saxion University of Applied Sciences, Deventer were included as a control group for the Dutch dental student population.

This study is reported in accordance with the STROBE guidelines for reporting observational studies [14]. The study was conducted in accordance with the principles of the 64^th^ WMA Declaration of Helsinki (October 2013, Brazil) and the Medical Research Involving Human Subjects Act (WMO), approximating Good Clinical Practice (CPMP/ICH/135/95) guidelines, and the study was approved by the Medical Ethical Committee (MEC) of the VU Medical Centre, Amsterdam, the Netherlands (2014.429).

### Study population and sample selection

As no previous valid information regarding the MDRB carriage rate was available, a non-probability sampling method was chosen. Within each dental school a convenience sample of clinical (STP+) and pre-/non-clinical (STP-) dental students was selected. Only students from the participating universities and over 18 years were eligible to participate in this study. Students were asked to participate in the study by the researchers, and not by teaching staff to ensure voluntary participation. Potential participants received a letter with information about the study and the study procedures. After adequate time to consider participation and to ask questions, written informed consent was obtained. Samples were coded, but not linked to the participants’ identity to assure that they would not face a study-delay when testing positive for MRSA (according to Dutch Ethical Guidelines). In compliance to the guidelines of the MEC, participants were able to be informed about their MRSA carriage by providing their sample number.

### Study procedures

Each participant received a questionnaire to collect demographic data. Questions on antibiotic use, hospital visits, patient treatment status and living in the vicinity of a livestock were asked to assess possible cofounders. After completion of the survey form, the students were carefully instructed on how to take the clinical sample. Three sites, each with a separate sterile cotton swab (Sarstedt, Nümbrecht, Germany), were sampled: (1) the interdigital folds between the ring- and little-finger on their dominant hand, (2) both anterior nares of their nose and (3) the dorsum of their tongue. Samples were immediately transported to a microbiology laboratory, and cultured in 500 µl Tryptic Soy Broth (TSB, BD, Sparks Glencoe, MD, USA). Cultures were stored at −80°C and stored until further analyses after the addition of 500 µl 60% (v/v) glycerol (Merck, Darmstadt, Germany). All culturing on solid media and in TSB were performed under aerobic conditions at 37°C for either 24 or 16 hours, respectively.

### MRSA

To assess the presence of MRSA in the stored samples, an aliquot of each sample was plated onto Mannitol-Salt Agar (MSA) and incubated. Single colonies with a yellow halo were identified as possible *S. aureus* and subcultured in TSB. To confirm antibiotic resistance, 10 µl of each overnight culture was added to the wells of microtiter plates containing 90 µl of TSB with various concentrations of oxacillin (0, 1, 2 and 4 μg/ml, Sigma, St. Louis, Mo, USA) and incubated subsequently. Isolates positive for growth on MSA and in TSB with 4 μg/ml oxacillin were selected and subcultured in TSB. *S. aureus* identity was confirmed by a positive coagulase test (Sigma, according to the manufacturer’s instruction). To assess the final identity of MRSA, genomic DNA was isolated from each positive isolate using the GeneJet kit (Thermo Fisher Scientific, Waltham, MA, USA). Isolated DNA was stored at −20°C, until further use. Possible MRSA strains, being positive for the presence of the *SSCmec* genes (*MecA* and *MecC*), were confirmed by PCR [15]. The MRSA results from Rome were published previously [16].

### ESBL and VRE

The presence of ESBL and VRE was established by plating aliquots of the frozen samples onto either ESBL CHROMagar^TM^ or VRE CHROMagar^TM^ plates, according to the manufacturer’s instructions (Biotrading Benelux, Mijdrecht, The Netherlands). Colonies resembling the proper morphology (according to instructions) were picked and streaked onto Blood Agar plates (40 gr/L Tryptic Soy Agar, 2 gr/L glucose, 5% defibrinated sheep blood) to check their purity. Each isolate was Gram’s stained and was subsequently subcultured in TSB. Each ESBL positive culture was spread onto Tryptic Soy Agar (BD) and incubated with an E-test® ESBL (BioMérieux Benelux B.V., Zaltbommel, The Netherlands) to confirm resistance towards cefepime (MIC ≥ 1.0 μg/ml) in the presence and absence of clavulanic acid [17]. VRE positives isolates were confirmed using an MIC-test towards vancomycin (0–16 mg/L vancomycin).

### Statistical analyses

The main study parameters were the presence of MSRA, ESBL, and VRE in students, associated with their contact with patients or other possible sources for multi-resistant bacteria. For each university, carriage rates of MDRB were assessed in both clinical and non-clinical students. The 95% confidence intervals (CI) were assessed using the Wilson method, with continuity correction, in order to account for relatively small sample sizes. To account for different sample sizes the overall weighted carriage rate was assessed, using the inverse of the variance as weight. The crude odds ratios (OR) for MRSA, ESBL, VRE, and any MDRB carriage in STP+ relatively to STP- were assessed. The overall weighted crude ORs were also assessed using the inverse of the variance weighting method. Additionally, the adjusted OR for MDRB carriage, attributable to clinical activity, was assessed using multiple logistic regression analyses, with a correction for study centres. Differences between students who treated patients and students who did not treat patients were tested using a Fisher exact test. Alpha was set at 0.05. Correction for multiple comparisons was performed using the Bonferroni correction; alpha was set on 0.025.

## Results

### Study population

A total of 879 students participated in this study; 454 students who treated patients (STP+) and 425 students who did not treat patients (STP-). In Thessaloniki, 196 students took part in the study (98 STP+), in Rome, 157 students (90 STP+), in The Netherlands 340 students (163 STP+) and in Gothenburg, 186 students (103 STP+). The demographic characteristics were different between the two groups; 64% of the STP+ were females compared to 73% females in the STP- group. The mean age of the STP+ (24.3 years ± 4.4) was not significantly higher compared to the STP- (20.1 years ± 3.0).

### MDRB carriage

A total of 50 students (5.7%) tested positive for an MDRB; 13 (1.5%) students tested positive for MRSA, 26 (3.0%) for ESBL, and 12 (1.4%) for VRE. One Swedish student tested positive for both ESBL and VRE, all other students tested positive for only one MDRB. The difference in prevalence between STP+ and STP- did not result in statistically significant differences for any of the MDRB and study centres, excluding MRSA carriage in the Italian student population which was significantly lower in STP+ than in STP- (Table 1). Eight students tested positive for an MDRB on more than one sampling site, of whom two students tested positive for MRSA on all three sampling sites (Table 2). All the crude OR estimates for MDRB carriage in STP+ relatively to STP- were not significant with wide CIs (Table 3), the point estimates ranged between 0.07 (95% CI 0.00–6.50 for MRSA in Italian students) and 4.43 (95% CI 0.49–40.03 for MRSA in Dutch students). The cumulative ORs were also not significant. The multiple regression analysis corroborated the latter result, as the adjusted OR for any MDRB resulted 1.11 (95% CI, 0.62–1.96) (Table 4). The only variable that was significantly associated to MDRB carriage was the recent use of antibiotics (OR 2.01; 95% CI, 1.10–3.68).

**Table 1.** Prevalence (with 95% CI between brackets) of MDRB (expressed in % per group per country) for students distributed by their clinical experience

**MDRB**	**Country**	**All students in %** (95% CI)	**STP+ in % (**95% CI)	**STP- in %** (95% CI)
**MRSA**	Thessaloniki	1.53 (0.40–4.77)	1.02 (0.05–6.36)	2.04 (0.35–7.89)
	Rome	3.18 (1.18–7.66)	0 (0.00–5.10)*	7.46 (2.78–17.25)*
	The Netherlands	1.47 (0.54–3.60)	2.45 (0.79–6.56)	0.56 (0.03–3.58)
	Gothenburg	0 (0.00–2.52)	0 (0.00–4.48)	0.00 (0.00–5.51)
	**Total (weighted)**	1.44 (0.76–2.46)	1.15 (0.39–2.61)	1.70 (0.70–3.42)
**ESBL**	Thessaloniki	4.08 (1.91–8.17)	4.08 (1.31–10.71)	4.08 (1.31–10.71)
	Rome	5.73 (2.82–10.93)	7.78 (3.45–15.89)	2.99 (0.52–11.32)
	The Netherlands	2.06 (0.91–4.38)	1.84 (0.48–5.71)	2.26 (0.73–6.06)
	Gothenburg	1.08 (0.19–4.25)	1.94 (0.34–7.52)	0 (0.00–5.51)
	**Total (weighted)**	2.96 (1.94–4.30)	3.63 (2.11–5.78)	2.44 (1.20–4.38)
**VRE**	Thessaloniki	1.53 (0.40–4.77)	0 (0.00–4.70)	3.06 (0.79–9.33)
	Rome	3.82 (1.56–8.50)	4.44 (1.43–11.62)	2.99 (0.52–11.32)
	The Netherlands	0.29 (0.01–1.88)	0.61 (0.03–3.88)	0.00 (0.00–2.65)
	Gothenburg	1.08 (0.19–4.25)	1.94 (0.34–7.52)	0.00 (0.00–5.51)
	**Total (weighted)**	1.34 (0.69–2.34)	1.54 (0.62–3.14)	1.00 (0.29–2.47)

The percentages per multi-drug resistant bacterium (MDRB) are weighted: percentage of positive student relative to the total of students corrected for the different numbers of participants per country. STP+ are students who treated patients (n = 454); STP- are students who did not treat patients (n = 425). Overall, and within centre differences between clinical and preclinical, students are not significant at 95% level, with one exception: * statistically significant difference p = 0.013.

**Table 2.** Frequency of MDRB on the sampling sites and in different countries

	**MRSA**				**ESBL**				**VRE**			
	Hand	Mouth	Nose	Total* (%)	Hand	Mouth	Nose	Total* (%)	Hand	Mouth	Nose	Total* (%)
Thessaloniki (n = 196)	1	1	3	**3 (1.5)**	4	0	4	**8 (4.1)**	1	0	2	**3 (1.5)**
Rome (n = 157)	3	3	2	**5 (3.2)**	4	5	4	**9 (5.7)**	2	2	2	**6 (3.8)**
The Netherlands (n = 340)	3	0	3	**5 (1.5)**	3	4	0	**7 (2.1)**	1	0	0	**1 (0.3)**
Gothenburg (n = 186)	0	0	0	**0 (0.0)**	0	2	0	**2 (1.1)**	1	1	0	**2 (1.1)**

MDRB are given as the number of students testing positive, per sampling site. Some students were positive for MDRB on more than one sampling site, which explains that some totals are not the sum of the previous columns. Percentages are given per country and per sampling site.

**Table 3.** Intra-centre and overall sample Odds ratios for MDRB carriage among clinical students (STP+) relative to nonclinical students (STP-) (weighted OR, 95% CI)

**Centre**	**MRSA**	**ESBL**	**VRE**	**All MDRB**
Thessaloniki	0.49 (0.04–5.55)	1.00 (0.24–4.12)	0.14 (0.01–2.72)	0.53 (0.17–1.65)
The Netherlands	4.43 (0.49–40.03)	0.81 (0.18–3.68)	3.28 (0.13–81.01)	1.77 (0.57–5.54)
Gothenburg	0.81 (0.02–41.09)	4.11 (0.20–86.87)	4.11 (0.20–86.87)	5.82 (0.30–114.2)
Rome	0.07 (0.00–6.50)	2.74 (0.55–13.64)	1.51 (0.27–8.51)	0.90 (0.35–2.31)
**Overall (weighted)**	0.76 (0.20–2.89)	1.45 (0.66–3.22)	1.13 (0.40–3.21)	1.01 (0.56–1.84)

All the odds ratios estimates were statistically not significant.

**Table 4.** Multiple logistic regression analyses to assess risk factors of MDRB carriage

Variables	OR (95%CI)	p-value	Reference group
Gender	1.00 (0.54–1.86)	0.99	Male
Age	0.99 (0.92–1.06)	0.80	Integral variable
Clinical experience	1.11 (0.62–1.96)	0.73	STP-
Antibiotics last year	2.01 (1.10–3.68)	0.02	‘No’
Working/living with livestock	1.22 (0.54–2.72)	0.63	‘No’
Hospitalization last year in foreign country	0.77 (0.22–2.74)	0.68	‘No’
Hours since last hand hygiene moment	0.89 (0.75–1.06)	0.20	Integral variable

All ORs were corrected for the four centres.

The prevalence of MDRB was statistically significantly different between countries (χ^2^ = 23.3, df = 3, p = 0.00004). *Post hoc* analyses revealed that Dutch students had significantly less MDRB cases as compared to Italian students (χ^2^ = 13.8, df = 1, p = 0.0002), Swedish students had significantly less MDRB cases as compared to Italian (χ^2^ = 16.8, df = 1, p = 0.00004) and Greek (χ^2^ = 6.86, df = 1, p = 0.009) students.

## Discussion

The prevalence of MRSA, VRE and ESBL-bacteria in this study was low in all centres and clinical activity was not significantly associated to any of the tested MDRB (Table 3), although MRSA prevalence was significantly lower in the Italian clinical students (Table 1). The only parameter associated with carriage of MDRB was the use of antibiotics the year before testing with 2.0 higher odds of carrying an MDRB in students who used antibiotics, although a causal relationship will have to be studied in a longitudinal study design.

In the EU/EEA, large differences in antibiotic use exist [13; 18], with the highest population-weighted mean consumption of antibiotics in Thessaloniki and the lowest in The Netherlands. The low carriage rates of MDRB found in the current study, even in Rome and Thessaloniki, were therefore unexpected. It should be noted that general prevalence data of these bacteria are often calculated as a ratio compared to the not-drug resistant species, with scarce data being available from the whole population. In larger epidemiological studies,, the carriage rates for MRSA, in the general population is 0% in Gothenburg, 0.8% in The Netherlands and 0.2% in Rome [13,19,20]. Even though the carriage rate seemed somewhat higher in Thessaloniki, it was not comparable with the 5.5% carriage reported previously [21].

As expected, the Swedish students did not carry any MRSA and had low carriage rates for VRE and ESBL-bacteria, which is in line with the prevalence data of MDRB of the Swedisch general population. No clear explanation was however, found for the difference between Roman students who treated patients compared to those who did not treat patients (0% vs 7.5%). The frequency of hand hygiene performance, of the students who treat patients, is suggested to be the basis for this difference.

Since Thessaloniki and Rome have a relatively high carriage rate of MDRB in the general population [13], the low prevalence amongst dental students was unexpected, and would suggest that frequent transmission of MDRB during patient treatment is not likely. However, the reference numbers from literature are based on an older and less healthy population compared to the current study participants.

Carriage rates for MRSA, in dentistry, differ considerably between countries (0–20%) [12,21–25]. These differences may be explained by differences in (the frequency of) antibiotic use per country and the data from this study does support this association. Other possible differences in application and compliance of infection control protocols can play a significant role in the management of MRSA transmission and other MDRB [21]. Unfortunately, reports on these topics, for different countries, are not available and would require international multi-centre studies to further explain our data in relation to previous studies. Regardless of the local MRSA prevalence within DHCPs, surface disinfection is an important step to prevent spreading MRSA within the dental clinic [24,26]. No previous studies report on the prevalence of ESBL and VRE in dentistry, but the current data suggest that the prevalence is not elevated amongst dental students.

The current study included considerably larger numbers of participants as compared to previous studies, providing an important contribution to a reliable overview of risks of transmission between people in the dental practice. It therewith fulfills the call from Yoo, et al [27,28], for multi-centre and multi-national studies on MRSA in dentistry. The current study could be prone to bias due to the convenience sampling in both the study and control group [29,30], may result in a different prevalence than the actual situation. However, our results may give an indication that the risk for MDRB carriage and the sequential transmission of MDRB for DHCPs and their patients is acceptably low in Europe, and may contribute to a better estimation of the risk of cross-contamination in dentistry.

However, multinational and multi-institu-tional studies are required to clarify the true MRSA carriage rate among DHCPs because most of the stud-ies were performed at a single institutio
